# Machine learning to predict in-stent stenosis after Pipeline embolization device placement

**DOI:** 10.3389/fneur.2022.912984

**Published:** 2022-09-06

**Authors:** Dachao Wei, Dingwei Deng, Siming Gui, Wei You, Junqiang Feng, Xiangyu Meng, Xiheng Chen, Jian Lv, Yudi Tang, Ting Chen, Peng Liu

**Affiliations:** ^1^Department of Interventional Neuroradiology, Beijing Neurosurgical Institute, Capital Medical University, Beijing, China; ^2^School of Biomedical Engineering, Capital Medical University, Beijing, China; ^3^Department of Interventional Neuroradiology, Beijing Tiantan Hospital, Beijing, China

**Keywords:** machine learning, flow diverter, Pipeline embolization device, complication, endovascular treatment, intracranial aneurysm

## Abstract

**Background:**

The Pipeline embolization device (PED) is a flow diverter used to treat intracranial aneurysms. In-stent stenosis (ISS) is a common complication of PED placement that can affect long-term outcome. This study aimed to establish a feasible, effective, and reliable model to predict ISS using machine learning methodology.

**Methods:**

We retrospectively examined clinical, laboratory, and imaging data obtained from 435 patients with intracranial aneurysms who underwent PED placement in our center. Aneurysm morphological measurements were manually measured on pre- and posttreatment imaging studies by three experienced neurointerventionalists. ISS was defined as stenosis rate >50% within the PED. We compared the performance of five machine learning algorithms (elastic net (ENT), support vector machine, Xgboost, Gaussian Naïve Bayes, and random forest) in predicting ISS. Shapley additive explanation was applied to provide an explanation for the predictions.

**Results:**

A total of 69 ISS cases (15.2%) were identified. Six predictors of ISS (age, obesity, balloon angioplasty, internal carotid artery location, neck ratio, and coefficient of variation of red cell volume distribution width) were identified. The ENT model had the best predictive performance with a mean area under the receiver operating characteristic curve of 0.709 (95% confidence interval [CI], 0.697–0.721), mean sensitivity of 77.9% (95% CI, 75.1–80.6%), and mean specificity of 63.4% (95% CI, 60.8–65.9%) in Monte Carlo cross-validation. Shapley additive explanation analysis showed that internal carotid artery location was the most important predictor of ISS.

**Conclusion:**

Our machine learning model can predict ISS after PED placement for treatment of intracranial aneurysms and has the potential to improve patient outcomes.

## Introduction

Flow diversion are widely used in the treatment of intracranial aneurysms. Among the various available devices, the Pipeline embolization device (PED; Medtronic, Dublin, Ireland) is the most widely studied. The PED was initially developed and approved for treatment of large and giant aneurysms located on the internal carotid artery (ICA) from the petrous to the superior hypophyseal segments (1). Owing to its high occlusion rate and satisfactory safety profile, PED use has been expanded to treat ruptured and unruptured saccular and non-saccular aneurysms of the anterior communicating, middle cerebral, vertebrobasilar, and posterior inferior cerebellar arteries (2–8). In-stent stenosis (ISS) is a common complication of PED placement and has been defined as intimal hyperplasia within the stent that appears as an unfilled contrast space between the contrast filled vascular cavity and stent on digital subtraction angiography (7). However, long-term complications of PED placement are not well understood. Previous studies have reported that most patients with ISS are asymptomatic and that ISS usually gradually improves; however, it may worsen (8–14). In addition, ISS may result in hemiplegia (14, 15) or even death (11) and cause decreased blood flow velocity (16). Considering that severe stenosis can progress to vascular occlusion and weaken the compensatory ability of the cerebral vasculature, its potential harm cannot be ignored. Therefore, the pathogenesis and predictors of ISS should be studied to improve long-term outcomes.

Previous retrospective studies have shown that balloon angioplasty (17), current smoking (18), prior cerebrovascular stenosis (18), dual antiplatelet therapy non-compliance (13), and anterior circulation location (13) are risk factors for ISS. Increasing year of treatment within the study period was also a risk factor in one study (17). Protective factors include increasing age (17), previous endovascular treatment (17), and statin use (11). However, a comprehensive ISS prediction model has not been developed for patients undergoing PED placement. This study aimed to establish a feasible, effective, and reliable ISS prediction model based on patient clinical and imaging characteristics using machine learning methods. Application of such a model can identify patients at high risk for ISS and enable close follow-up, which should improve long-term outcomes.

## Materials and methods

### Study population

**Figure 1:**
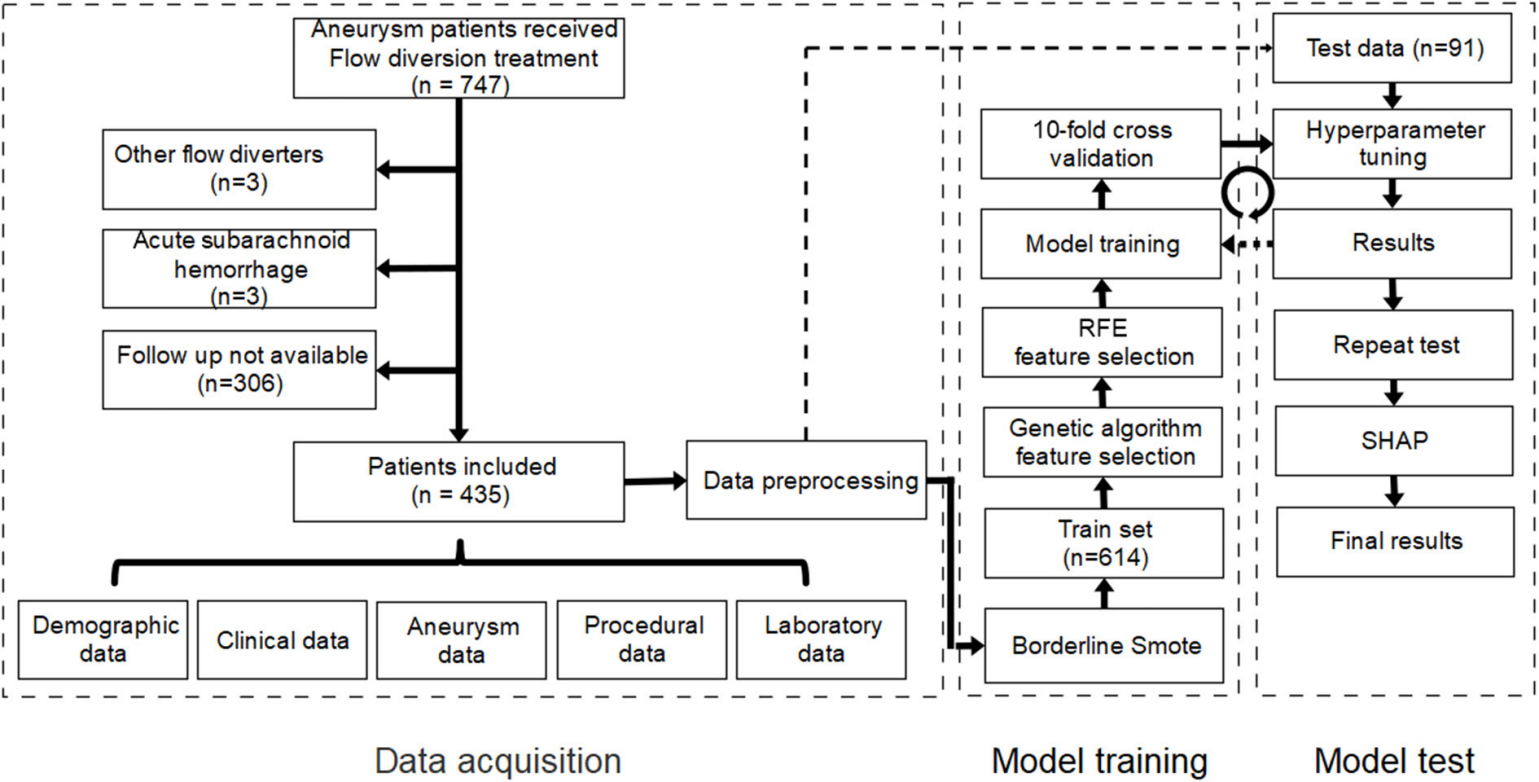
Study flow chart of patient selection and establishment of the machine learning model. RFE, recursive feature elimination; SHAP, Shapley additive explanation.

Data for patients treated with flow diverters in the Department of Interventional Neuroradiology, Beijing Tiantan Hospital between January 2015 and October 2020 were retrospectively collected. Only patients treated using the PED who had at least one angiographic follow-up were eligible for study inclusion. In our center, patients scheduled for implantation of PED was administered with aspirin (100 mg) and clopidogrel (75 mg) for at least 5 days prior to the procedure. And the duration of dual antiplatelet therapy ranged from 3 to >6 months after procedure, and a combination of aspirin (100 mg/day) and clopidogrel (75 mg/day) was the most common antiplatelet regimen. We excluded patients who had experienced subarachnoid hemorrhage within 1 month prior to PED placement and those whose imaging studies before or after treatment were not available. Institutional review board approval was obtained. The requirement for informed consent was waived because the study was retrospective in nature and all data were deidentified. A study flow chart of patient selection is illustrated in Figure 1.

### Data collection

We collected and recorded clinical and laboratory data from the electronic medical records and reviewed imaging studies (digital subtraction angiography, computed tomography angiography, magnetic resonance angiography) performed before and after treatment. Perioperative laboratory data included data from 14 days before to 14 days after treatment. Imaging follow up was performed 6 and 12 months after treatment and every year thereafter for 5 years. If a laboratory parameter had multiple recordings, the mean value was recorded. Aneurysm morphological parameters, including maximum diameter, neck diameter, maximum height, perpendicular height, aneurysm width, aspect ratio, size ratio, height/width ratio, neck ratio, and bottleneck factor, were manually measured by three experienced neurointerventionalists according to previously published studies (19, 20) (Supplementary Table 1). Parent artery diameter, proximal parent artery diameter, and distal parent artery diameter (defined as the minimum diameter of the parent artery at the aneurysm neck, 1.5 × parent artery diameter upstream from the neck, and 1.5 × parent artery diameter downstream from the neck, respectively) were measured manually at the same point in the imaging studies before and after treatment (21). Stenosis rate was calculated according to the formula:


$$\begin{array}{l} Stenosis\;Rate\;\left(SR\right)=\\ 1\;-\;\frac{parent\;artery\;diameter\;at\;certain\;follow\;up\;(D_{x})}{intraopeartive\;parent\;artery\;diameter\;(D_{0})\;} \end{array}$$

ISS was defined as stenosis rate >50% within the PED. ISS was graded as mild (50–74%), severe (75–99%), or occlusion (100%). Aneurysm occlusion was graded according to the O'Kelly-Marotta (OKM) grading scale (22), which is based on the degree of aneurysmal filling: Total filling, subtotal filling, entry remnant, or no filling.

### Data preprocessing

Among the 122 variables recorded, 92 were included for analysis after excluding those in which >30% of values were missing (Supplementary Table 2). Missing values were imputed using the random forest method in the missingpy package (version 0.2.0). Continuous variables were standardized using z-score transformation. Categorical variables were binarized. Multicategorical variables were converted into binary variables using one-hot encoding.

The processed dataset was randomly stratified into training (80%) and test (20%) sets. A bias toward negative cases was present because of the scarcity of patients with ISS. Therefore, borderline-SMOTE was applied to the training set using the imblearn package (version 0.8.0). This technique can generate synthetic data from the minority class (patients with ISS) to achieve balance of negative and positive cases (23). After application of borderline-SMOTE, the training set was expanded to 614 cases (307 stenosis cases).

### Feature selection and model training

We applied and compared five popular machine learning models: elastic net (ENT), support vector machine (SVM), Xgboost (XGB), Gaussian Naïve Bayes (GNB), and random forest (RF) with traditional logistics regression (LR) using the open-source machine learning library scikit-learn (version 0.24.1). Before model training, genetic algorithm (GA) and recursive feature elimination (RFE) were each applied to the training set to identify the best combination of features. Then, 10-fold cross validation and grid search were used in model training to determine the optimal hyperparameters of each model. The performance of the machine learning models was evaluated using sensitivity, specificity, and area under the receiver operating characteristic curve (AUC-ROC) in the test set. The flow chart for model training and testing is shown in Figure 1.

After model training and testing, we applied Monte Carlo cross-validation (MCCV) to verify the efficacy of the machine learning model again. The dataset was randomly divided into test and training sets and the training and testing were repeated 100 times. Sensitivity, specificity, AUC-ROC, maximum Youden index, and threshold at maximum Youden index in each loop were recorded. Mean sensitivity, mean specificity, and mean AUC-ROC were calculated to determine model performance. Mean value of maximum Youden index in each loop was calculated and determined as the optimal threshold.

### Model explanation

The Shapley additive explanation (SHAP) algorithm (version 0.39.0) was used to address interpretability problems associated with machine learning models. Based on game theory, SHAP connects optimal credit allocation with local explanations using the classic Shapley values. SHAP can simultaneously provide local and global model interpretation (24).

### Statistical methods

Statistical analyses were performed using Python (version 3.8.8). Categorical variables are expressed as numbers with percentage. Continuous variables with normal distribution are expressed as means ± standard deviation; those with skewed distribution are expressed as medians with interquartile range (IQR). Normality was tested using the Shapiro–Wilk test. One-way analysis of variance was used to compare Monte Carlo cross-validation between the machine learning models. The *post hoc* Tukey honestly significant difference (HSD) test was applied to identify where the differences lay. The highest Youden's index was used to define the optimal cut-off value. The mean value of the optimal cut-off value was used to differentiate low and high stenosis risk. The association between stenosis risk and time after procedure was assessed using Cox regression. The log-rank test was then used to compare Kaplan–Meier curves. Two-tailed *P* ≤ 0.05 was considered significant.

## Results

### Study population and stratified random sampling

Based on our inclusion criteria, 435 patients were finally enrolled. Two hundred and eighty-nine (66.4%) were female. Median age was 54 years (IQR, 47–61). Average body mass index (BMI) was 24.9 (IQR, 22.7–26.7). Sixty-seven patients had BMI >28. One hundred and eighty-four patients (42.3%) had a history of hypertension; 19 (4.4%) had a history of subarachnoid hemorrhage. Seventy-one patients were current or former smokers. Ninety-three aneurysms were non-saccular. Aneurysm location was ICA in 335, vertebral artery in 86, basilar artery 12, middle cerebral artery in 10, and other in 10. Average aneurysm size and neck width were 12.97 ± 8.17 mm and 8.98 ± 6.24 mm, respectively. As of July 2021, 69 ISS cases (15.2%) had been identified; follow-up was available in 66. Among these, 20 (30.3%) were symptomatic. Symptoms included moderate to severe headache (9/20), dizziness or vertigo (5/20), contralateral limb movement disorder (3/20), visual impairment (2/20), neurological deficit (1/20), visual field defect (1/20), and cognitive impairment (1/20). Poor outcome (modified Rankin scale score ≥3) was experienced by five patients (7.6%): one ocular motility disorder, two ISS-related deaths, and two deaths unrelated to ISS (one aneurysm rupture and one acute myocardial infarction).

Random stratification of the cohort resulted in placement of 614 patients (307 stenosis cases) in the training set and 91 (14 stenosis cases) in the test set.

### Feature selection

To find the best combination of characteristics, a GA-based program was developed and used; three iterations were performed over the 92 variables in the training set to yield nine predictors (age, obesity, balloon angioplasty, operation duration, size ratio, neck ratio, ICA location, platelet-large cell ratio, and red cell volume distribution width [RDW-CV]). Then we applied the RFE algorithm to the training set and identified 12 predictors (age, height, weight, BMI, obesity, recurrent aneurysm, balloon angioplasty, aneurysm morphology, bifurcation location, ICA location, neck ratio, RDW-CV). Finally, we used the six common features (age, obesity, balloon angioplasty, ICA location, neck ratio, and RDW-CV) in GA and RFE to train the model.

### Cross validation and hyperparameter tuning

**Figure 2:**
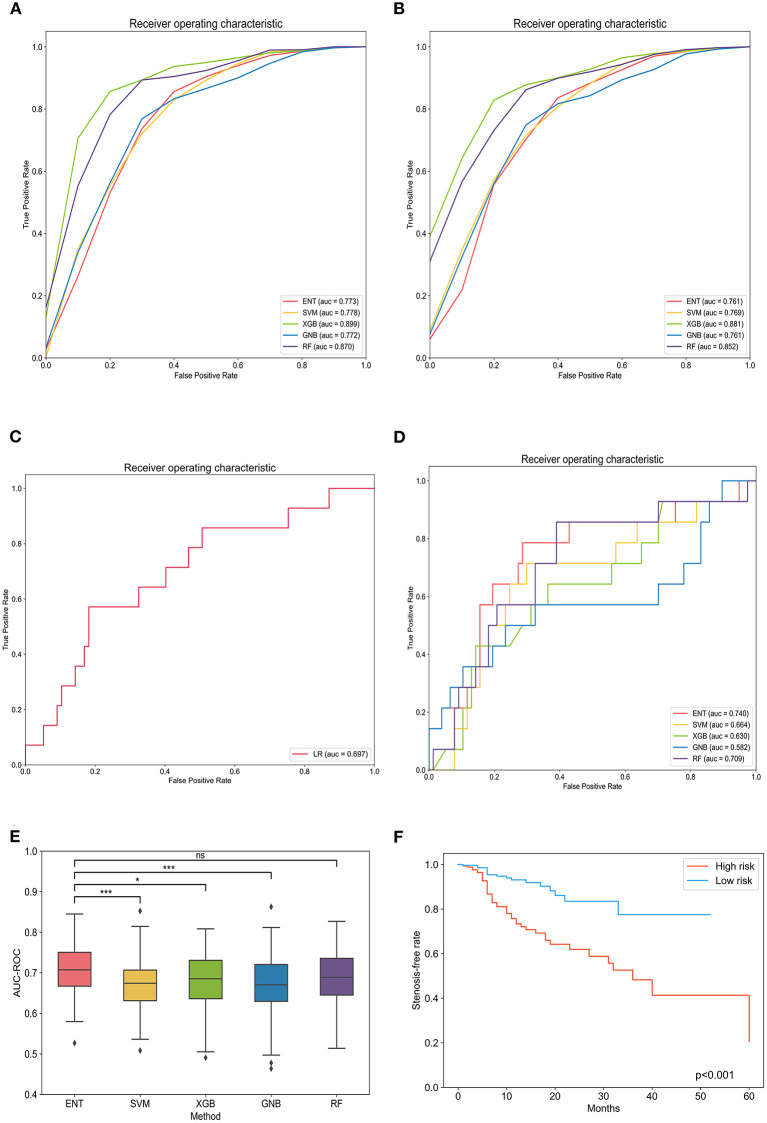
Evaluation of machine learning model performance in the training, validation, and test sets. **(A)** Comparison of the area under the receiver operating curve of different models in the training set. **(B)** Comparison of the area under the receiver operating curve of different models in the validation set. **(C)** Comparison of the area under the receiver operating curve of different models in the test set. **(D)** The receiver operating curve of logistics regression. **(E)** Box plot of model area under the receiver operating curves in each loop. *Tukey honestly significant difference (HSD) test *p* < 0.05 between the models; ^***^Tukey HSD test *p* < 0.005 between the models; ^ns^Tukey HSD test *p* > 0.05 between the models. **(F)** Kaplan–Meier curves of in-stent stenosis rates for high-risk patients (predicted value > optimal threshold) and low-risk patients (predicted value < optimal threshold). ENT, elastic net; SVM, support vector machine; XGB, Xgboost; GNB, Gaussian Naïve Bayes; RF, random forest; LR, logistics regression.

After 10-fold cross validation and hyperparameter tuning, the best hyperparameters were identified. Model performance is illustrated in Figures 2A,B. In the training set, the XGB model had the highest mean AUC-ROC (0.899; 95% confidence interval [CI], 0.897–0.902), followed by the RF model (0.870; 95% CI, 0.868–0.871), SVM model (0.778; 95% CI, 0.775–0.780), ENT model (0.773; 95% CI, 0.769–0.776), and GNB model (0.772; 95% CI, 0.768–0.775). In the validation set, the XGB model also had the best mean AUC-ROC (0.881; 95% CI, 0.861–0.900), followed by the RF model (0.852; 95% CI, 0.831–0.874), SVM model (0.769; 0.742–0.797), ENT model (0.761; 95% CI, 0.733–0.790), and GNB model (0.761; 95% CI, 0.736–0.785). Then, we tested the models in the test set (Figure 2C). We also tested the performance of logistics regression (LR) (Figure 2D). Though the performance was inferior in cross validation, the ENT model had the highest AUC-ROC in the test set (0.740), followed by the RF model (0.709), SVM model (0.664), XGB model (0.630) and GNB model (0.582). LR had an AUC-ROC of 0.697, which was lower than ENT and RF. The confusion matrix was shown in Table 1. ENT model is a combination of lasso regression and ridge regression, which add regular terms to logistics regression to avoid overfitting. Given that, we believed that ENT model is better than LR and can represent the performance of LR.

**Table 1:** Confusion matrix of models in the test set.

**Model name**		**ENT**		**SVM**		**XGB**		**GNB**		**RF**	
		**Stenosis**	**No stenosis**	**Stenosis**	**No stenosis**	**Stenosis**	**No stenosis**	**Stenosis**	**No stenosis**	**Stenosis**	**No stenosis**
True	Stenosis	9	5	6	8	7	7	9	5	6	8
value	No stenosis	20	57	20	57	20	57	36	41	14	63

ENT, elastic net; SVM, support vector machine; XGB, Xgboost; GNB, Gaussian Naïve Bayes; RF, random forest. Green: the prediction of models is consistent with the true value. Red: the prediction of models is inconsistent with the true value.

To exclude the influence of randomness in the process of assigning patients to the training and test sets, we applied Monte Carlo cross-validation and recorded the AUC-ROC, best Youden index, thresholds at best Youden index, and corresponding sensitivity and specificity in each loop. The ENT model remained the optimal model (0.709; 95% CI, 0.697–0.721) with a mean sensitivity of 77.9% (95% CI, 75.1%−80.6%) and specificity of 63.4% (95% CI, 60.8%−65.9%), followed by the RF model (0.687; 95% CI, 0.674–0.700), XGB model (0.680; 95% CI, 0.668–0.693), GNB model (0.675; 95% CI, 0.661–0.689), and SVM model (0.670; 95% CI, 0.657–0.683; Table 2). One-way analysis of variance and Tukey HSD multiple comparison showed that the ENT model's mean AUC-ROC significantly outperformed the SVM model, XGB model, and GNB model (*p* = 0.001, *p* = 0.018, *p* = 0.003, respectively); however, the mean AUC-ROC did not significantly differ between the ENT and RF models (p = 0.131; Figure 2E).

**Table 2:** Comparison of model performance in the training, validation, and test sets.

**Model name**	**Training**			**Validation**			**Test**		**Monte Carlo cross-validation**											
	**Mean** **AUC**	**95% CI**		**Mean** **AUC**	**95% CI**		**AUC**	**Accuracy**	**Mean** **AUC**	**95% CI**		**Mean** **sensitivity**	**95% CI**		**Mean** **specificity**	**95% CI**		**Mean** **accuracy**	**95% CI**	
		**Low**	**High**		**Low**	**High**				**Low**	**High**		**Low**	**High**		**Low**	**High**		**Low**	**High**
ENT	0.773	0.769	0.776	0.761	0.733	0.790	0.740	0.725	0.709	0.697	0.721	0.779	0.751	0.806	0.634	0.608	0.659	0.666	0.646	0.686
SVM	0.778	0.775	0.780	0.769	0.742	0.797	0.664	0.692	0.670	0.657	0.683	0.758	0.733	0.782	0.624	0.599	0.649	0.641	0.623	0.658
XGB	0.899	0.897	0.902	0.881	0.861	0.900	0.630	0.703	0.680	0.668	0.693	0.719	0.691	0.747	0.662	0.634	0.690	0.669	0.649	0.689
GNB	0.772	0.768	0.775	0.761	0.736	0.785	0.582	0.549	0.675	0.661	0.689	0.673	0.644	0.702	0.707	0.683	0.732	0.699	0.683	0.716
RF	0.870	0.868	0.871	0.852	0.831	0.874	0.709	0.769	0.687	0.674	0.700	0.722	0.693	0.751	0.666	0.640	0.692	0.651	0.633	0.669

AUC, area under the curve; CI, confidence interval; ENT, elastic net; SVM, support vector machine; XGB, Xgboost; GNB, Gaussian Naïve Bayes; RF, random forest.

### Model explanation

Spearman correlation testing showed a significant positive correlation between the predicted scores and degree of stenosis (*r* = 0.418, *p* < 0.001). The ENT model was applied to all patients to obtain predicted scores. All patients were grouped according to risk (low-risk and high-risk groups) according to the optimal threshold determined in Monte Carlo cross-validation of Cox regression analysis. Cox regression showed that ISS risk was significantly higher in the high-risk group than the low-risk group (hazard ratio 3.41; 95% CI, 2.03–5.73, *p* < 0.001; Figure 2F).

**Figure 3:**
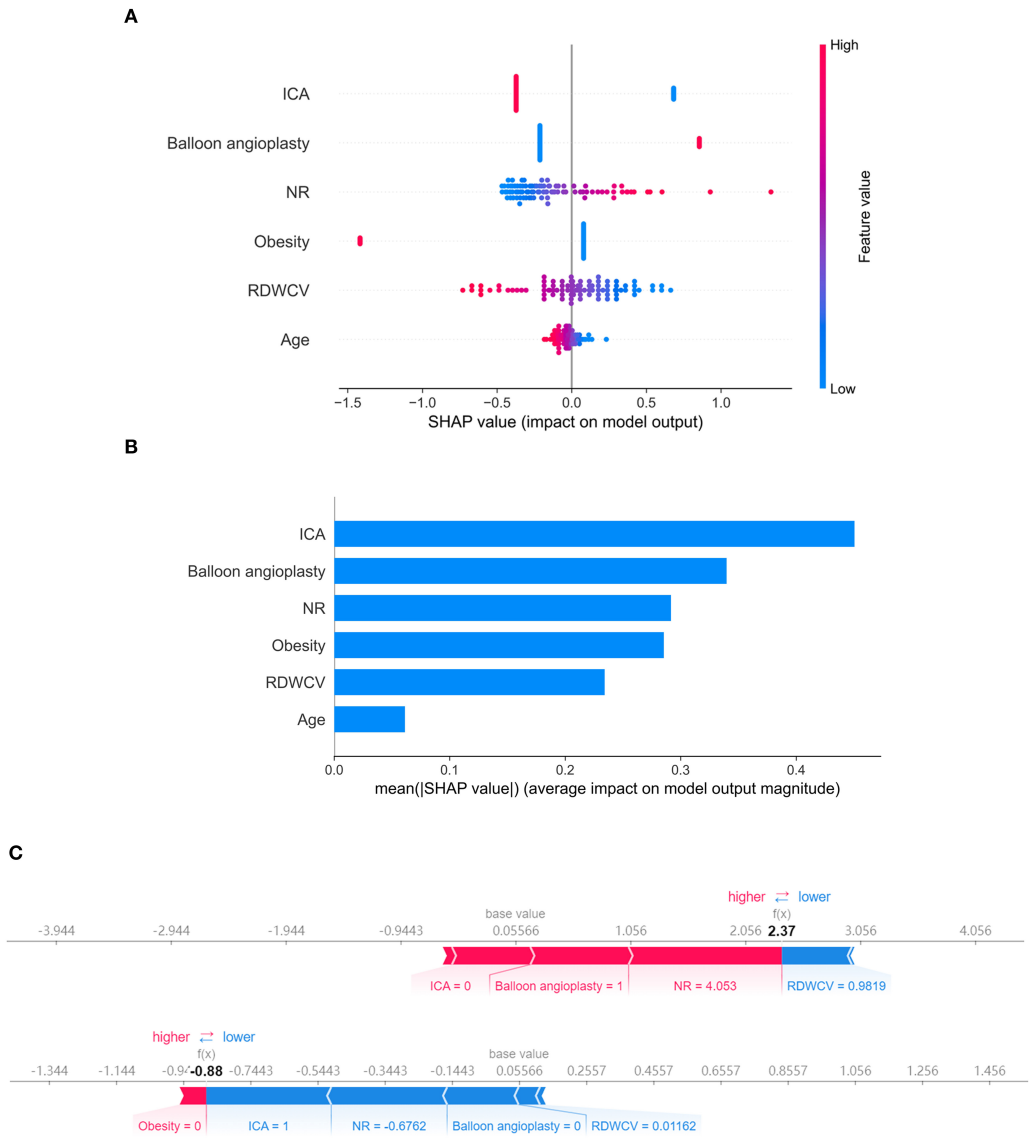
Shapley additive explanation (SHAP) analysis of the elastic net (ENT) model. **(A)** Association between the SHAP value and feature value. **(B)** Feature importance (mean |SHAP value|) of each predictor. **(C)** Two ENT model prediction examples. ICA, internal carotid artery; NR, neck ratio; RDWCV, coefficient of variation of red cell volume distribution width.

Next, we used SHAP analysis to interpret the ENT model. Figure 3A shows the importance of the different variables. ICA location had the greatest influence on the model, followed by balloon angioplasty, neck ratio, obesity, RDW-CV, and age. Figure 3B shows the influence of feature values on model prediction. The X-axis represents the influence on the model (SHAP value), the right of the X-axis represents the positive influence, and the left of the X-axis represents the negative influence. The color of the point represents the value of the feature: red represents high feature value and blue represents low feature value. Therefore, balloon angioplasty and increasing neck ratio are risk factors for ISS, while ICA location, obesity, increasing RDW-CV, and increasing age are protective factors. Figure 3C shows the interpretation of SHAP analysis for two individual patients. Case 1 is a patient without ISS in whom the model correctly predicted no stenosis. The influence of various factors on model prediction is shown in the figure. Case 2 is a patient with ISS in whom the model correctly predicted stenosis.

## Discussion

We developed a machine learning-based prediction model that can predict ISS in intracranial aneurysm patients who undergo PED placement. Six factors predict ISS: ICA location, balloon angioplasty, neck ratio, obesity, RDW-CV, and age. Among the five machine learning models, the ENT model had best performance as measured by AUC-ROC, sensitivity, and specificity. Moreover, the result of Monte Carlo cross-validation strongly demonstrated the efficacy and robustness of the machine learning model. We also found a positive correlation between predicted scores and ISS grade. Using the optimum threshold from Monte Carlo cross-validation, we stratified patients according to risk of ISS and showed that the model's risk stratification was accurate. Finally, we utilized SHAP analysis to perform explanations for the machine learning model.

To our knowledge, this is the first prediction model to predict ISS in patients with intracranial aneurysms treated using a flow diverter. ISS is a common complication of flow diverter placement. Sweid et al. reported a 6.3% incidence and noted that ISS was the most common complication (17). A meta-analysis reported an 8.8% incidence (8). In our study, ISS caused symptoms in 30.3% of affected patients and 7.6% experienced a poor outcome. However, most had a good outcome and most patients with ISS were asymptomatic. In addition, ISS in most patients remain stable or even improved. These findings are consistent with previous studies (8–14). Reversible stenosis may be associated with thrombosis (25). Flores-Milan et al. reported an ISS-related death from a stroke secondary to cerebral artery occlusion (11). It remains unclear whether delayed thrombosis, ISS, and patient symptoms are related.

In view of the high incidence and potential harms of ISS, predicting its occurrence, identifying risk factors, and stratifying patients according to risk are necessary to enable better patient care and prevent complications. The ability to predict ISS would enable preoperative evaluation of postoperative risk, which would assist treatment decision making. Furthermore, in patients with low risk of ISS, unnecessary follow-up could be avoided, while high-risk patients would be closely observed and treated appropriately to reduce the risk of acute ischemic complications.

In contrast with the traditional and regular machine-learning based prediction models, our model has several advantages, namely identification of six ISS predictors, use of Borderline-SMOTE in model training, and use of feature selection. We identified six predictors of ISS: ICA location, obesity, increasing RDW-CV, and increasing age were protective factors, while balloon angioplasty and increasing neck ratio were risk factors. Predictors found in previous studies are consistent with ours. Brinjikji et al. (26) found a trend toward higher ISS rates among younger patients; all ISS cases in their study occurred in patients under 50 years of age (2/793). Sweid et al. (17) reported that increasing age is negatively associated with ISS (odds ratio 0.9; *p* = 0.02). Higher rates of ISS in younger individuals have also been reported in stent-assisted coiling and coronary artery stenting studies (27–29); these higher rates have been attributed to more intense intimal hyperplasia within the device in younger individuals. Sweid et al. (17) also reported balloon angioplasty as an ISS predictor (odds ratio 4.2; p = 0.03). John et al. (9) found a higher rate of balloon angioplasty in ISS patients (40 vs. 2%), but they did not conduct statistical inference because of the small number of cases. Balloon angioplasty may result in endothelial damage that induces intimal hyperplasia. This hyperplasia may then progress and eventually cause ISS. In our study, ICA position was negatively associated with ISS, which contradicts the results of Chalouhi et al. (13). The inconsistency may be due to confounding factors. In our cohort, aneurysms in the posterior circulation were mostly fusiform, and those located on the ICA were saccular. Therefore, confounding of location and morphology may have been present. Notedly, Potts et al. (14) reported that fusiform morphology is an ISS predictor for aneurysms in the anterior circulation. Srinivasan et al. (10) reported similar findings. The fact that fusiform aneurysms may need a longer construct or placement of multiple overlapping devices may explain this, as either may cause more damage to the vascular endothelium. Moreover, fusiform aneurysms tend to have a larger neck width, which take longer to completely endothelialize. Increasing neck ratio was an ISS risk factor in our study, which is in agreement with the findings of Potts et al. (14) Interestingly, obesity (BMI >28) was protective against ISS. In a previous percutaneous coronary intervention meta-analysis, West et al. (30) reported that lower BMI (*p* = 0.04) was associated with restenosis, which is in accordance with our findings. Although obese patients in our study had larger artery diameter than patients with BMI <28, the difference was not significant (3.90 vs. 3.69 mm; *p* = 0.10, Mann–Whitney U-test). Future studies should elucidate the reason for this finding and explore the relationship between BMI, arterial diameter, and ISS risk. Our study found increasing RDW-CV was a protective factor, which has not been previously reported. We do not yet know the exact mechanism linking RDW-CV and ISS; however, removing RDW-CV from the model will cause a 0.02–0.05 decrease in AUC-ROC. Further work is required to establish the validity of RDW-CV in ISS prediction.

Datasets in classification of diseases or complications are often imbalanced between the numbers of negative and positive cases. Because models based on such datasets may be inaccurate, balancing methods should be implemented. The application of Borderline-SMOTE in our study significantly improved model performance in predicting positive cases; however, it “forged” some positive cases in the strive to balance, which could be controversial in medicine. Therefore, we only used Borderline-SMOTE in the training set; real test data was used to validate the model in model testing.

Feature selection is an important process in machine learning. Selecting the proper combination of features to achieve a balance between model performance and efficiency is difficult but of great significance. Classical methods of feature selection, such as filter-based methods, which include univariate regression, variance threshold, and maximal information coefficient, have difficulty solving multicollinearity. Therefore, we developed a GA-based feature selection program. A GA simulates the progress of biological evolution. It starts with some chromosomes and individuals (representing a possible combination of features), evaluates the fitness of individuals (AUC-ROC of the validation set), and selects individuals with better fitness to survive, while others will be mutated or crossed over. This process continues until fitness improvement is below the threshold or the maximum number of iterations is reached. In principle, a GA is a random search algorithm. It is possible that it finds a solution that is optimal locally but not globally that is adequate for predicting. We entered 92 variables, iterated over them, and obtained a combination of nine variables. RFE was further used to validate the genetic algorithm results. RFE is a greedy algorithm in essence. It can also achieve a locally optimal solution by removing the most unimportant features repeatedly until the desired number of features is reached. After RFE, there were 12 remaining features, some of which coincided with the GA algorithm, thus verifying the reliability of the GA algorithm. Finally, we used six common features of RFE and GA results to train the model.

Our study has several limitations. First, the study was retrospective in design and conducted in a single center, which may limit the generalizability of our model. A multicenter prospective study is needed in the future for model validation. Second, our dataset had a relatively low number of ISS patients. Although we used Borderline-SMOTE to address this problem, better model performance could be achieved if more ISS cases were available. Third, stenosis measurement was manual and based on different angiographic imaging modalities; therefore, measurement error may have been introduced. However, the mean values of measurements obtained by three different neurointerventionalists were used. In the future, application of deep learning to aneurysm morphology measurement may reduce such errors. Fourth, because of the large number of missing values, we removed all variables in which >30% of the values were missing and used the random forest method to impute missing values in the remaining variables. Fifth, machine learning models are difficult to interpret, which limits their application in medicine. We used SHAP to further illustrate our results. SHAP analysis can provide an explanation for every prediction, which can help clinicians understand model decision making and facilitate application of machine learning models. Sixth, we did not include the length and the diameter of PED in the model because of data deficiency. Longer stent has larger area of contact between the stent and the blood vessels which may result in more damage to the vascular endothelium. Seventh, Exclusion of patients with subarachnoid hemorrhage may weaken the generalization of the results. A subgroup analysis between ruptured aneurysms and unruptured ones may help solve the problem, but we did not have sufficient data.

## Conclusion

Our machine learning model can predict ISS after PED placement for treatment of intracranial aneurysms and has the potential to improve patient outcomes.

## Data availability statement

The raw data supporting the conclusions of this article will be made available by the authors, without undue reservation.

## Ethics statement

The studies involving human participants were reviewed and approved by IRB of Beijing Tiantan Hospital Affiliated to Capital Medical University. Written informed consent from the participants' legal guardian/next of kin was not required to participate in this study in accordance with the National Legislation and the Institutional requirements.

## Author contributions

DW, DD, SG, and WY were involved in conception and design of the study. DW, DD, and SG organized the database and performed data analysis. DW drafted the manuscript. WY, JF, XM, XC, JL, YT, TC, and PL critically revised the manuscript and approved its final version. All authors contributed to the article and approved the submitted version.

## Funding

This study was supported by the National Key Research and Development Program of China (grant No. 2017YFB1304400) and the Youth Program of the National Natural Science Foundation of China (grant No. 81901197).

## Conflict of interest

The authors declare that the research was conducted in the absence of any commercial or financial relationships that could be construed as a potential conflict of interest.

## Publisher's note

All claims expressed in this article are solely those of the authors and do not necessarily represent those of their affiliated organizations, or those of the publisher, the editors and the reviewers. Any product that may be evaluated in this article, or claim that may be made by its manufacturer, is not guaranteed or endorsed by the publisher.
