# Supplementary material for: Machine learning to predict in-stent stenosis after Pipeline embolization device placement
Source: Front Neurol. 2022 Sep 6;13:912984. doi: 10.3389/fneur.2022.912984 (PMC9486156; doi:10.3389/fneur.2022.912984)
Supplement: Supplementary file 1 [file Table_1.DOCX]

Supplementary Material

# Supplementary Tables

## Supplement Table 1. Definitions of Morphological parameters

| **Morphological parameter** | **Definition** |
| --- | --- |
| Maximum diameter | Largest distance between any 2 points on the aneurysm surface that could be fitted inside the dome |
| Neck width | The longest diameter of the neck plane |
| Maximum height | The largest distance between the neck center and aneurysm surface that could be fitted inside the dome |
| Perpendicular height | The largest orthogonal distance from the neck to the aneurysm dome |
| Aneurysm width | The largest distance orthogonal to the maximal height |
| Parent artery diameter | minimum diameter of the parent artery at the aneurysm neck |
| Proximal artery diameter | 1.5 × parent artery diameter upstream from the neck |
| Distal artery diameter | 1.5 × parent artery diameter downstream from the neck |
| Aspect ratio | Maximum height/neck width |
| Height/width ratio | Perpendicular height/aneurysm width |
| Size ratio | Maximum diameter/parent artery diameter |
| Neck ratio | Neck width/parent artery diameter |
| Bottleneck factor | Neck width/aneurysm width |
| Diameter difference | proximal artery diameter - distal artery diameter |
| Diameter ratio | proximal artery diameter / distal artery diameter |

## Supplement Table 2. 92 variables included in model training

| **Demographic Features** |
| --- |
| Gender |
| Age |
| height |
| weight |
| BMI |
| Obesity |
| **Medical history** |
| Diabetes |
| Dyslipidemia |
| Coronary artery disease |
| Hypertension |
| Previous SAH |
| Previous stroke |
| Allergy |
| Smoke |
| Alcohol consumption |
| **Aneurysm morphological parameter** |
| Multiple |
| Location |
| ICA |
| VA |
| Left or right |
| Anterior or posterior |
| Symptomatic |
| Aneurysm type |
| Bifurcation |
| Lobulation |
| Maximum diameter |
| Neck width |
| Maximum height |
| Perpendicular height |
| Aneurysm width |
| Parent artery diameter |
| proximal artery diameter |
| distal |
| Aspect ratio |
| Height/width ratio |
| Size ratio |
| Neck ratio |
| Bottleneck factor |
| Diameter difference |
| Diameter ratio |
| **Procedural information** |
| Recurrent aneurysm |
| Previous treatment |
| therapy |
| Number of PED |
| Operation duration |
| Balloon angioplasty |
| Improper stent placement |
| Postoperative complication |
| Aneurysm persistence |
| Occlusion |
| **Laboratory data** |
| ALT |
| AST |
| TP |
| ALB |
| GLB |
| A/G |
| GLU |
| CO2 |
| UA |
| K |
| Na |
| Cl |
| Ca |
| P |
| WBC |
| LY# |
| MONO# |
| NEUT# |
| EO# |
| BA# |
| EO% |
| BA% |
| RBC |
| HGB |
| HCT |
| MCV |
| MCH |
| MCHC |
| RDW |
| RDW-CV |
| PLT |
| PDW |
| MPV |
| P-LCR |
| PCT |
| PT(time) |
| PT(INR) |
| APTT(time) |
| Fbg |
| TT(time) |
| NLR |
| PLR |
| **Excluded variables** |
| SP |
| R |
| K |
| MA |
| TMA |
| EPL |
| TPI |
| G |
| LY30 |
| E |
| AA inhibition |
| ADP inhibition |
| TG |
| CHO |
| HDL |
| LDL |
| APO-A1 |
| APO-B |
| CREA |
| BUN |
| HBDH |
| CK |
| TBA |
| CHE |
| DBIL |
| TBIL |
| IBIL |
| LDH |
| ALP |
| GGT |
